# Prevalence and distribution of ampc beta-lactamase producing *escherichia coli* and *klebsiella pneumoniae* isolates obtained from urine samples at a tertiary care hospital in the caribbean

**DOI:** 10.3389/fcimb.2022.1015633

**Published:** 2022-10-18

**Authors:** Camille-Ann Thoms Rodriguez, Felecia Dawson, Jenene Cameron, Christine Seah, Marvin Reid, Roberto G. Melano, Maxine Gossell-Williams

**Affiliations:** ^1^ Department of Microbiology, The University of the West Indies, Kingston, Jamaica; ^2^ Department of Microbiology, The University Hospital of the West Indies, Kingston, Jamaica; ^3^ Department of Basic Medical Sciences, The University of the West Indies, Kingston, Jamaica; ^4^ Department of Clinical Lab and Microbiology Sciences, Public Health Ontario Laboratory, Toronto, ON, Canada; ^5^ Caribbean Institute for Health Research, The University of the West Indies, Kingston, Kingston, Jamaica; ^6^ Department of Laboratory Medicine and Pathobiology, Faculty of Medicine, University of Toronto, Toronto, ON, Canada

**Keywords:** AmpC, beta-lactamase, uropathogens, mdro, antibiotic resistance

## Abstract

**Introduction:**

The aim of this study was to investigate the prevalence and distribution of AmpC beta-lactamases (BLs) in uropathogens (*E. coli* and *K. pneumoniae*) at the University Hospital of the West Indies Jamaica (UHWI).

**Method:**

De-duplicated consecutive urine samples, collected from January to March 2020 at the UHWI, were analyzed. Screening and phenotypic confirmatory tests were conducted using resistance to cefoxitin and the Disc Approximation Test (DAT) respectively, for isolates of interest. Multiplex PCR was performed on cefoxitin resistant (CR) isolates for the detection of *bla*_CIT_, *bla*_MOX_, *bla*_FOX_, *bla*_ACC_, and *bla*_DHA_ genes. Whole genome sequencing (WGS) was used to further detect AmpC BL genes in PCR negative isolates with indeterminate phenotypic results.

**Results:**

Sixty-four Gram negative isolates were obtained from 61 patients (55% female), aged 18 months to 88 years old. At least 35% (26) had complicated urinary tract infections. Only 7 out of 64 isolates were *E. coli* or *K. pneumoniae*, had antibiograms suggestive of possible AmpC BL production and were CR. DATs confirmed AmpC BL in two of these (1 K*. pneumoniae*; 1 *E. coli*), one tested negative (*E. coli*) and four had inconclusive results (*K. pneumoniae).* PCR detected *bla*_DHA_ and *bla*_CIT_ in two CR isolates. WGS further detected *bla*_CMY-42_ in one isolate. The prevalence of screened CR isolates with AmpC BL is 57.14% (4 of 7), representing 6.25% of the sample. AmpC BL producers tested had 100% susceptibility to meropenem and nitrofurantoin.

**Conclusion:**

AmpC BL prevalence among *E. coli* and *K. pneumoniae*, common urinary pathogens, in the studied isolates is low. Although cefoxitin screening is helpful, phenotypic screening using the DAT can yield indeterminate results best clarified by molecular testing.

## Introduction

Antibiotic resistance has dramatically increased over the years endangering the lives of many patients. Several mechanisms, including the production of drug inactivating enzymes like the AmpC beta-lactamase (AmpC BL), are responsible. The production of drug inactivating enzymes is the main mechanism of resistance seen in Gram-negative bacteria (Ventola, 2015). Gram-negative organisms within the Enterobacteriaceae can possess intrinsic and acquired resistance mechanisms to beta-lactam antibiotics with varying spectra of activity. The production of BL such as AmpC BL however is among the most common mechanism of resistance (vs. target modification, efflux and permeability) that these organisms deploy to emerge resistant to beta-lactam antibiotics. AmpC BLs can hydrolyze penicillins and extended spectrum cephalosporins, and are uninhibited by clavulanic acid (Munita and Arias, 2016). Some bacterial species such as *E. coli* and *Klebsiella pneumoniae* can acquire AmpC BL genes through conjugative plasmids (pAmpC BL), and isolates such as these, which are among the most common causes of urinary tract infections, should be prioritized for testing for AmpC BLs. Infections caused by Gram-negative organisms which express AmpC BL are especially important as their presence limits available therapeutic options (Tamma et al., 2019). Current guidelines of the Infectious Diseases Society of America recommend that the drugs of choice for the treatment of serious infections caused by organisms producing AmpC BLs are the carbapenems (Harris and Ferguson, 2012).

A recent systematic review identified an increase in the worldwide prevalence of pAmpC BLs in the Gram negative organisms *Escherichia coli* and *Klebsiella pneumoniae* over the past ten years (Rodríguez-Guerrero et al., 2022). However data from the Caribbean was not represented in this study (Rodríguez-Guerrero et al., 2022). In a study commissioned among the Caribbean Public Health Agency member countries, AmpC BL genes were detected among the included isolates and the authors suggested a need for further studies to determine the prevalence of this mechanism of resistance in the region, as well as its effect on carbapenem sensitivity (Heinz et al., 2019).

*E. coli* and *Klebsiella* species are very important Gram-negative uropathogens, significantly featured in samples collected from 2002 to 2004, 2008 and 2012 at the University Hospital of the West Indies (UHWI). They had levels of resistance to penicillins and cephalosporins greater than 30%, but negligible evidence of resistance to carbapenems (Nicholson et al., 2009; Cheung et al., 2020). The aim of this study was to determine the prevalence and distribution of AmpC BL-producing uropathogens, specifically *E. coli* and *K. pneumoniae* at the UHWI, which is a major referral centre in the Caribbean. We describe the phenotypic and genotypic characteristics of such isolates.

## Materials and methods

### Research strategy

A retrospective descriptive cross-sectional study was conducted at the Department of Microbiology, the University of the West Indies (located at the UHWI), Mona, Jamaica, after ethical approval was sought and obtained from the UWI Mona Campus Research Ethics Committee. The UHWI is a 500-bed tertiary care referral centre for the Caribbean. It is located near the capital Kingston and receives referrals from across the island and wider Caribbean.

Sixty-four de-duplicated, archived clinical isolates were obtained from 61 patients from January to March 2020 and processed for identification by standard biochemical analyses and susceptibility testing using the Kirby Bauer disk diffusion in accordance with the CLSI guidelines (CLSI, 2021). An adequate sample size (using Cochran’s formula) for the study period was determined to be 64, at a 95% confidence interval and a precision of 5.6% using a prevalence of 8.5% determined after an audit of the files at the Department of Microbiology was conducted (Simeon and Picou, 2003). Recommendations for quality control testing were observed (CLSI, 2021).

All *E. coli* and *K. pneumoniae* isolates showing resistance to cefuroxime-sodium, ceftazidime and ceftriaxone were selected to undergo cefoxitin screening for the detection of AmpC BLs. Isolates that were resistant to cefoxitin were selected to undergo further confirmatory phenotypic and genotypic tests (PCR) and for some, whole genome sequencing (WGS) (Polsfuss et al., 2011).

### Disc approximation test (DAT)

The confirmatory phenotypic test used was the DAT. For this test, isolates resistant to cefoxitin where sub-cultured using blood agar plates, incubated for 18-24 hours for 35 (+/-2) °C. A 0.5 McFarland bacterial suspension was made from the incubated blood agar plates and swabbed on Mueller Hinton Agar (MHA) and allowed to dry. A ceftazidime disk (30µg) was placed in the centre of the plate, and disks of imipenem (10µg), cefoxitin (30µg), and clavulanate/amoxicillin (10/20µg) were placed 20 mm apart from the ceftazidime disk. Plates were inverted and incubated at 35 (+/- 2) °C for 18-24 hrs. A positive AmpC test was indicated by a blunting or flattening of the zone of inhibition between the ceftazidime disk and the surrounding substrates (imipenem, cefoxitin and clavulanate disk) (Gupta et al, 2014). Well characterized isolates recovered and verified in a Canadian teaching hospitals were used as positive controls. Along with positive controls, a negative control was prepared using *E. coli* ATCC 25922 and included in the test.

### Polymerase chain reaction and gel electrophoresis

Two multiplex PCR reactions were performed for detection of *bla*_CIT_, *bla*_MOX_, *bla*_FOX_, *bla*_ACC_, and *bla*_DHA_ (Polsfuss et al., 2011). **Table 1** shows the primer sequences used in this study (Liu and Liu, 2016). DNA of CR isolates was extracted by the boiling method. 1µL of the supernatant from the DNA extract was then added to 19µL of master mix made using 10µL Red Taq (Accuris ™), 3µL (triplex)/5µL (duplex) PCR water (Thermo Fisher Scientific) and 1µL of the primers (Integrated DNA Technologies) as outlined above. The PCR machine was set with an initial denaturation step at 94°C for 3min, followed by 25 cycles of DNA denaturation at 94°C for 30 sec. Primer annealing for 64°C for 30 sec and primer extension at 72°C for 1 min followed by final extension at 72°C for 7 min. The amplicons were visualized in an agarose gel following standard procedures. The same positive and negative controls used for the DAT were used for PCR.

**Table 1:** Primer sequences for target sites.

Gene	Primer		Amplicon Size
	Forward	Reverse	
*bla*_MOX_	5’ – GCT GCT CAA GGA GCA CAG GAT3’	5’– CAC ATT GAC ATA GGT GTG GTG C 3’	520
*bla*_FOX_	5’ – AAC ATG GGG TAT CAG GGA GAT G 3’	5’ – CAA AGC GCG TAA CCG GAT TGG 3’	190
*bla*_CIT_	MF5’ – TGG CCA GAA CTG ACA GGC AAA3’	MR 5’ – TTT CTC CTG AAC GTG GCT GGC ‘3	462
*bla*_DHA_	5’ – AAC TTT CAC AGG TGT GCT GGG T 3’	5’ CCG TAC GCA TAC TGG CTT TGC 3’	405
*bla*_ACC_	5’ – AAC AGC CTC AGC AGC CGG TTA 3’	5’ – TTC GCC GCA ATC ATC CCT AGC 3’	346

### Wgs

Isolates that showed an inconclusive DAT result and were negative by multiplex PCR underwent WGS using the Illumina platform. DNA was extracted using the MasterPure Complete DNA & RNA Purification kit (Epicenter Illumina, Wisconsin USA) with elution carried out to a final volume of 40 μL in TE buffer. Sequencing libraries and runs were performed as previously described (Gianecini et al., 2019). Resistance genes were detected using the StarAMR pipeline (https://github.com/phac-nml/staramr), which scans genome contigs against the ResFinder, PlasmidFinder, and PointFinder databases (Center for Genomic Epidemiology). The genome sequences of the isolates included in this study were deposited in GenBank and were registered under BioProject accession number PRJNA861980.

AmpC BL prevalence was determined using the results of all the phenotypic and genotypic tests, to give as an accurate report as possible.

## Results

Of the 61 subjects most were female (33 versus 25 males; data was missing for 3 subjects) aged 1 month to 93 years old (mean 44.5 years; data was missing for 1 subject). Among those for whom clinical data was obtained (79%), 48.97% were diagnosed with uncomplicated UTIs. The 64 isolates were identified as *E. coli* (45.31%), *K. pneumoniae* (34.38%), *Proteus mirabilis* (7.81%), *Klebsiella aerogenes* (6.25%), *Salmonella* Group D (2.35%), and *Enterobacter* spp., *Proteus vulgaris* and *Panteoa agglomerans* (1.56% each). Antimicrobial susceptibility data suggested that the antibiotic which most isolates were susceptible was meropenem, followed by amikacin, gentamicin and nitrofurantoin. Only 21 out of 64 isolates had antibiograms suggestive of possible AmpC BLs production with resistance to amoxicillin-clavulanate, ceftazidime, and ceftriaxone. These isolates were *E. coli* (n=8) and *K. pneumoniae* (n= 13). Among these 21 isolates, seven (33.33%) were CR (five *K. pneumoniae* and two *E. coli*). Only 2 isolates were confirmed as AmpC BL producers following the use of the DAT for confirmation (**Table 2**). This gives a prevalence of at least 28.57% using this phenotypic test among the CR isolates. The results obtained for four of the remaining isolates were inconclusive and required additional confirmatory tests. The remaining isolate was negative. Multiplex PCR analyses revealed that only two CR isolates had AmpC BL genes, *bla*_CIT_ and *bla*_DHA_ (**Table 2**) giving a similar prevalence using PCR only. Isolates that gave inconclusive phenotypic results and were negative by PCR underwent WGS and among these only one was found to have a AmpC BL gene, *bla*_CMY-42_, which was not detected because specific primers for blaCMY were not included in the molecular screening used. Interestingly, two isolates that gave inconclusive results were found *via* WGS to have the *bla*_NDM-5_ gene. The overall prevalence of AmpC BL among CR *E. coli* and *K. pneumonia*e isolates within the sample is 57.14% (4 of 7). This represents 6.25% of the entire sample. AmpC BL producers had 100% susceptibility to meropenem and nitrofurantoin.

**Table 2:** Phenotypic and genotypic analysis of the seven cefoxitin resistant (CR) isolates for AmpC BL.

Organism	Phenotypic Results (Disk Approximation Test)	Genotypic Results	Meropenem Susceptibility Results
*K. pnuemoniae*	Inconclusive	Negative	Resistant
*K. pnuemoniae*	Inconclusive	*bla*_CIT_ +ve	Susceptible
*K. pnuemoniae*	+	*bla*_DHA_ +ve	Susceptible
*E. coli*	+	Negative	Susceptible
*K. pnuemoniae*	Inconclusive	Negative	Resistant
*E. coli*	–	Negative	Susceptible
*K. pnuemoniae*	Inconclusive	*bla*_CMY-42_+ve	Susceptible
*Positive* Control	+	*bla*_CIT_ +ve	N/A
*Negative* Control	–	Negative	N/A

N/A: 'Not Applicable'.

## Discussion

To our knowledge, this study is the first report detailing phenotypic and genotypic detection of AmpC BLs among urinary isolates in Jamaica along with associated clinical findings. While there have been reports primarily on the molecular detection of AmpC BL genes, there was less emphasis on the phenotypic detection and associated clinical characteristics as described in our study (Heinz et al. (2019) (Ashiboe-Mensah et al., 2016). With increasing resistance to antibiotics globally, we sought to further highlight challenges with detection in resource limited settings and add to the literature data on the types of genes seen in our geographical location. This will help in the further understanding of the dissemination of these resistance genes with movement to and from this popular tourist destination.

We noted that only 7 isolates of our sample were known to harbor inducible chromosomal AmpC (cAmpC) enzymes. Among the remaining 57 isolates only 7 met the criteria for further investigation as these were the isolates that were found to be CR. Overall, AmpC BL prevalence in CR uropathogens at UHWI was 57.14%. However, the overall prevalence of CR Gram negative bacteria without inducible cAmpC was low (10.94%). Among the isolates that did not carry inducible chromosomal genes the AmpC BLs prevalence rate was (7.02%).

Infection prevention and control measures are important strategies needed to limit the spread of these organisms and should be critical in all health-care settings. It was further noted that patients with complicated urinary tract infections were among those more likely to be affected for which, further investigations are needed in order to determine the reasons for this. In this regard, an evaluation of antibiotic usage would be helpful.

The use of molecular testing strategies such as multiplex PCRs are currently the gold standard for the detection of AmpC BL genes (Oliveira et al., 2019). Multiplex PCR analyses indicated that only two isolates were positive for *bla*_CIT_ and *bla*_DHA_. WGS sequencing also detected *bla*_CMY-42_. *bla*_CMY_ is the most predominant plasmid mediated AmpC gene in among certain uropathogens, in particular *E. coli* (Jacoby, 2009). Future studies focused on these pathogen are required to determine the AmpC gene prevalence in the country. Song et al. (2006), reported that *bla*_DHA_, which is an inducible AmpC BL gene, was most predominant in the *Enterobacteriaceae* in areas such as Korea and Japan. According to Black et al. (2005) the inducible *bla*_DHA_ gene would be more clinically harmful than others as it had a higher crude mortality rate than other genes for example *bla*_CMY-42_. Therefore, the *bla*_DHA_ is of concern. Ongoing surveillance is needed to monitor any further emergence of isolates with this resistance mechanism so that appropriate mitigation strategies can be employed. The *bla*_CIT_ gene which was amplified is known to have a higher frequency in regions such as Spain, China, India, Turkey and Egypt having been reported mostly in organism such as *K. pneumoniae* and *E. coli* (Rizi et al., 2020). The results in this study indicated AmpC genes were not detected in 71.4% of the CR isolates which is consistent with findings reported previously (Rizi et al., 2020). Possible reasons for this include deficiencies or mutations in associated genes or the presence of other BLs such as extended-spectrum beta-lactamases and/or carbapenemases. It could have also been due to the presence of a gene not covered under the primer pairs included in the study.

The high susceptibility to meropenem should not be taken for granted as carbapenemases among other mechanisms of carbapenem resistance continue to emerge globally and threaten the utility of this antibiotic. Interestingly, the two *K. pneumoniae* isolates that were cefoxitin and meropenem resistant (**Table 2**) were found to have the *bla*_NDM-5_ gene which further underscores this point. Antimicrobial strategies aimed at preventing the misuse of this antibiotic thereby guarding its shelf life is critical particularly in this resource limited setting where antimicrobial options are very few, leaving almost no alternatives for treatment. These would include nitrofurantoin, ciprofloxacin and cefepime, but unfortunately a high level of resistance is already being reported for the latter two in this sample. All the isolates that were CR and had susceptibility to cefepime, tested were also cefepime resistant.

The detection of AmpC BL in clinical settings continue to pose a problem as there still remains a paucity of guidelines detailing the types of methodologies that should be used. The lack of standardized tests for their detection by the CLSI have allowed many institutions to use the recommended guidelines by researchers (Polsfuss et al., 2011). There are challenges with some of the available phenotypic tests and the genotypic ones are often expensive and not routinely available in resource limited countries like Jamaica. Using the cefoxitin disk to screen for AmpC BL can be difficult because ACC-like enzymes produce larger inhibition zones, resulting in false negatives screens and so an alternative method of screening may need to be devised (Oliveira et al., 2019). It is therefore important to note that using phenotypic screening in this manner may limit the subsequent detection of genes associated with AmpC BL production as samples may contain bacteria that are reservoirs of resistance genes that do not necessarily express them under the conditions of the phenotypic profile study. This is a noted limitation of this study. Cefoxitin screening however helps to eliminate unnecessary tests which is important for resource limited settings. Thus far, there are several confirmatory phenotypic tests for detection including Disc Potentiation Test, Double Disc Synergy Test, AmpC Disc Test, Disc Approximation Test (DAT), along with the incorporation of inhibitors such as boronic acid or cloxacillin (Rodríguez-Guerrero et al., 2022). The cefoxitin screened isolates in our study underwent DAT and four had inconclusive results. This may be due to interference caused by coproduction of other BLs that affect the detection of AmpC activity, such as carbapenemases, and low-level expression of the AmpC gene which would make it difficult to detect using this method. Interestingly, Al-Marzooq et al. (2015), indicated that the DAT is particularly successful for the detection of *bla*_DHA_ plasmid gene in *K. pneumoniae*. We were therefore not surprised to see that one of the isolates that yielded a positive DAT result contained the *bla*_DHA_ gene. Given the possibility of a false negative phenotypic result, however, we concur with multiplex PCR being recommended as the gold standard method for AmpC detection. There is still need for more sensitive and specific phenotypic tests that will pick up AmpC BLs that are affordable and accessible in resource limited settings. Resource limitations prevented the performance of more comprehensive molecular analyses in this study and is one of the study limitations.

The limitations of this study included the fact that this was a single centre study making the findings not generalizable to the rest of the population. It is still noteworthy to have documented the detection of these enzymes. An expanded study looking at other infections would have also been helpful. Another limitation is a lack of data on clonality. WGS of all isolates would also provide further insights into the other types of resistance genes these plasmids were found to carry. It is hoped that this can be pursued in further studies. This study focused on the detection of AmpC BLs gene in CR Gram negative uropathogens (*E. coli and K. pneumoniae*), however other possible mechanisms of cefoxitin resistance like porin loss mutants could not be evaluated. With the AmpC BL gene “ACC” having larger zones of inhibition to cefoxitin, a false negative result may arise from cefoxitin screening. Some authors do however recommend cefoxitin screening in order to minimize unnecessary testing and optimize resources. This is perhaps an area for further study. It would also have been good to include other more sensitive and reliable phenotypic tests which have now replaced the DAT. The advantage of using the DAT in a resource limited setting however, is that it utilizes materials that would have likely been available and in use for routines tests. Further work could include WGS for further evaluation and screening of all cephalosporin resistant Gram-negative bacteria for the AmpC gene.

## Conclusion

A low prevalence of AmpC BL is being reported among certain uropathogens from UHWI Jamaica, with only 3 gene types detected (*bla*_CIT_, *bla*_DHA_ & *bla*_CMY-42_). Surveillance is needed to follow any further emergence of these AmpC BLs. All isolates with AmpC BLs were susceptible to meropenem, the drug of choice for serious infections.

## Data availability statement

The datasets presented in this study can be found in online repositories. The names of the repository/repositories and accession number(s) can be found below: GenBank and were registered under BioProject accession number PRJNA861980.

## Ethics statement

The studies involving human participants were reviewed and approved by The University of the West Indies, Mona Campus Research Ethics Committee. Written informed consent from the participants’ legal guardian/next of kin was not required to participate in this study in accordance with the national legislation and the institutional requirements.

## Author contributions

C-AT conceptualized the study, was involved in data analysis, data interpretation, drafting and finalizing the manuscript. FD was involved in data collection, data analysis, drafting and finalizing of the manuscript. JC was involved in data collection and analysis. CS and MR were involved in data analysis/interpretation. RM and MG-W was involved in data analysis and interpretation and revised the manuscript critically for intellectual content. All authors approved the final version of the manuscript and agree to be accountable for the work.

## Acknowledgments

We acknowledge the efforts of and support from Prof. Tony Mazzulli, the Mount Sinai Hospital Canada, the Department of Microbiology, UWI, Mona, the Dockets Department at the University Hospital of the West Indies, the Basic Medical Sciences Department at the University of the West Indies and Public Health Agency Canada.

## Conflict of interest

The authors declare that the research was conducted in the absence of any commercial or financial relationships that could be construed as a potential conflict of interest.

## Publisher’s note

All claims expressed in this article are solely those of the authors and do not necessarily represent those of their affiliated organizations, or those of the publisher, the editors and the reviewers. Any product that may be evaluated in this article, or claim that may be made by its manufacturer, is not guaranteed or endorsed by the publisher.
